# A Methodological Approach to Quantifying Silent Pauses, Speech Rate, and Articulation Rate across Distinct Narrative Tasks: Introducing the Connected Speech Analysis Protocol (CSAP)

**DOI:** 10.3390/brainsci14050466

**Published:** 2024-05-07

**Authors:** Georgia Angelopoulou, Dimitrios Kasselimis, Dionysios Goutsos, Constantin Potagas

**Affiliations:** 1 Neuropsychology & Language Disorders Unit, 1st Neurology Department, Eginition Hospital, Faculty of Medicine, National and Kapodistrian University of Athens, 115 28 Athens, Greece; georginangel@gmail.com (G.A.); dkasselimis@gmail.com (D.K.); 2 Department of Psychology, Panteion University of Social and Political Sciences, 176 71 Athens, Greece; 3 Department of Linguistics, School of Philosophy, National and Kapodistrian University of Athens, 106 79 Athens, Greece

**Keywords:** connected speech, silent pauses, speech rate, articulation rate

## Abstract

The examination of connected speech may serve as a valuable tool for exploring speech output in both healthy speakers and individuals with language disorders. Numerous studies incorporate various fluency and silence measures into their analyses to investigate speech output patterns in different populations, along with the underlying cognitive processes that occur while speaking. However, methodological inconsistencies across existing studies pose challenges in comparing their results. In the current study, we introduce CSAP (Connected Speech Analysis Protocol), which is a specific methodological approach to investigate fluency metrics, such as articulation rate and speech rate, as well as silence measures, including silent pauses’ frequency and duration. We emphasize the importance of employing a comprehensive set of measures within a specific methodological framework to better understand speech output patterns. Additionally, we advocate for the use of distinct narrative tasks for a thorough investigation of speech output in different conditions. We provide an example of data on which we implement CSAP to showcase the proposed pipeline. In conclusion, CSAP offers a comprehensive framework for investigating speech output patterns, incorporating fluency metrics and silence measures in distinct narrative tasks, thus allowing a detailed quantification of connected speech in both healthy and clinical populations. We emphasize the significance of adopting a unified methodological approach in connected speech studies, enabling the integration of results for more robust and generalizable conclusions.

## 1. Introduction

Connected speech, meaning the ability to organize speech output in text level, following a specific semantic context, as for example a picture description or a story narration [1], is considered as one of the most appropriate tools for investigating speech beyond the word level, as in picture-naming tasks. Despite the inherent challenge of quantifying connected speech output, using several numeric indices, such as speech rate as words per minute, speakers’ responses are presumed to closely approximate their communicative abilities in everyday life. Thus, the exploration of connected speech offers information of high ecological validity, surpassing the insights gained from standardized neuropsychological assessment, such as picture-naming tasks [1]. In language research, connected speech can offer valuable resources for studying both healthy speakers and individuals with developmental or acquired language deficits. Several microstructure and macrostructure linguistic elements can be derived from the implementation of elicitation techniques in distinct speech genres, offering insights into speakers’ linguistic and cognitive abilities [2] (see Table 1). Although numerous studies have investigated speech output ability using connected speech, certain restrictions have also become apparent in both healthy speakers and individuals with language disorders. These mostly relate to variability in methodologies implemented across studies, which presents a challenge to the formulation of general conclusions. Moreover, focusing on isolated fluency metrics, e.g., speech rate or articulation, disregards the fact that each index offers unique information about individuals’ ability to successfully produce speech output. In the current study, we propose a specific methodological approach to analyzing connected speech that takes into account the several specific metrics of fluency.

### 1.1. Silent Pauses’ Measures

Various fluency metrics exist with regard to either speech output or silent gaps. Silent pauses, also referred to as empty or unfilled pauses, silent gaps, or hesitations in the relevant literature, are the empty gaps identified in speech flow. The occurrence of silent pauses has been studied within the framework of speech output derived either from elicitation tasks of connected speech or reading tasks, involving healthy speakers [29], patients with language disorders (acquired or developmental), and neurological or psychiatric entities with cognition deficits (see, for example, [22] for patients with post-stroke aphasia; [24] for patients with primary progressive aphasia; Pistono et al. [17] for patients with Alzheimer’s Disease; [25] for patients with schizophrenia). Silent pauses gradually came to the attention of linguists and psychologists, as it became clear that their appearance is associated with specific cognitive processes, such as word finding or speech output organization (for a discussion, see [22]).

Despite the growing interest in the investigation of silent pauses, the literature reveals several inconsistencies in the methodological approaches employed for their study. Among the earlier studies, pauses were distinguished into longer and shorter, a feature that implied distinct functions according to duration. Goldman-Eisler [29] suggested a specific threshold (approximately 250 ms) as an a priori criterion in order to separate silent pauses into two distinct categories and, consequently, into two different functions. Specifically, silent pauses of shorter length than 250 ms were thought to serve only phono-articulation purposes [30], such as motor programming of speech output and speech movement execution [31], while longer pauses were seen as reflecting more complex cognitive processes, such as selective retrieval and word-finding processes, along with sentences’ organization of semantic context and morphosyntactical structure (see also [7] for a discussion).

This methodological approach of adopting a predetermined threshold has been widely applied in the majority of studies on pauses; however, the exact value of this threshold has been arbitrarily defined, leading to studies defining longer pauses using several thresholds and, as a result, different time boundaries (for instance, in [16,24,27] researchers implemented a criterion greater than 200 ms; in [17] they used a 250 ms threshold; in [15] they applied a floating threshold of 200–250 ms). Specialists in the field of computational linguistics have raised concerns about the misleading nature of the threshold approach [32]. Instead, they advocate for methodological approaches in which all detectable pauses should be included in each analysis, regardless of their duration, without relying on predetermined thresholds. This would ensure that the dataset in question is complete and accurate. Data from pauses themselves would indicate whether a threshold actually exists and where this would be located in time flow, without applying predetermined values. In other words, a potential threshold separating pauses according to their duration is considered as “dynamic”, influenced by several factors, including speaker’s health, age, and sex, as well as the genre of elicitation technique [33]. Thus, a new approach has emerged in which all pauses are included in the analysis, aiming at identifying whether their duration is subject to different frequency distributions with specific time boundaries (see, for example, [22,23,32,34]). Despite this, recent studies, as previously mentioned, often still implement specific thresholds, leading to the exclusion of a significant number of silent pauses from investigation. This practice raises questions about the validity of comparisons with other studies or meta-analyses.

Another crucial issue pertains to the specific metrics employed by each study in the investigation of silent pauses. A thorough investigation of previous studies reveals that, in some cases, only frequency is reported (see, for example, [17,24]), usually referred to as pause rate, indicating the number of pauses per 100 words (see, for example [16,22]), while there are other cases in which pause rate indicates the number of pauses per minute (see, for example [13]) or per total duration [23]. Other studies have also examined duration expressed through various measures, such as mean (see, for example, [23,25,26]), median (see, for example, [17,22,23]) or total duration of silent pauses (see, for example, [14,15]). It is worth noting that, in the case of total duration, some authors choose to standardize the duration based on the total number of words (see, for example, [14]), while others are based on the total number of pauses [15].

A last issue concerns the combination of filled and silent pauses; while most contemporary studies differentiate between silent and filled pauses in their analysis (as seen in, for example [14,22,25]), there are still some studies that treat both categories as the same linguistic phenomenon (for instance, [24,27]). This ongoing controversy hinders attempts to delve into the precise role of silent phenomena in speech output.

In conclusion, there is a lack of consistency in the definition, calculation, and metrics used to analyze silent pauses across methodological approaches. This, coupled with the limited and sporadic research dedicated to patterns of silent pauses, raises concerns about the reliability of results and their comparability across studies, as the varied use of metrics, including frequency or duration, makes standardization challenging. Consequently, attempts at direct comparisons or meta-analyses may produce unreliable or inconclusive outcomes due to the inconsistency in measurement methodologies.

### 1.2. Speech Fluency Measures

The speed or rate of speaking, typically referred to as “speech rate” or “speaking rate” in contemporary studies, is one of the most common measures of speech fluency. This metric is frequently employed in studies of acquired language disorders (e.g., [9,18,28,35]). Well-documented literature also exists for healthy populations across various languages and dialects, with a predominant focus on the effects of age, sex, and years of formal schooling (see, for example, [3,4,36]). Speech rate usually concerns the total number of words or syllables uttered, divided by the total length of narration in minutes, encompassing the duration of silent and filled pauses during speech (see, e.g., [9,10] for studies in aphasia; [3] for a study in healthy speakers). It must be noted, however, that syllables are considered to be a more accurate and representative measure of speech rate, as speech samples may include words of varying length of syllables [20]. In contrast, articulation rate is a measurement referring to pure speaking rate and articulator displacement, meaning an index reflecting the amount of time spent in articulation process, excluding any parts of silent or filled pauses [20,37]. It corresponds to the total number of words or syllables divided by the duration of pure speaking, excluding any intervals and/or disfluencies such as silent or filled pauses (see [18] for a clear distinction between the two measures of fluency).

Despite this general rule, many contradictory definitions appear in the literature for both speech and articulation rate. As a result, a detailed description of the exact speech measurement and calculations used in each study is, in most cases, more helpful in making sense of the relevant results. For instance, DeDe and Salis [8] distinguish articulation measures into articulation rate, where the duration of silent pauses and breaths is excluded, but duration of filled pauses and false starts is included, and pure word rate, where the duration of all kinds of disfluencies is excluded. Apart from these methodological discrepancies, the importance of studying specific measures of speech fluency is unquestionable in the field of language research. Speech rate has been often treated as a biomarker for the diagnosis of neurological pathologies with and without language disorders, such as in the categorization of different primary progressive aphasia variants [14,38] and in some cases even for the detection of the early onset of certain diseases (see, for example, a scoping review of early Alzheimer’s disease and mild cognitive impairment, [39]). Nevertheless, only a few studies highlight the discrepancy between speech rate and articulation rate measures (see, for example, [12]). Moreover, there is increasing evidence that speech rate is a more complex measure of fluency ability, which should be carefully treated and preferably analyzed, taking into consideration the duration of articulation and pauses which seem to play a major role in its definition.

Goldman-Eisler [40] was one of the pioneering researchers who provided evidence that speech rate is influenced by the duration of pauses and, as a result, should be calculated separately from articulation rate. She investigated speech rate, articulation rate (referred to as “absolute speech rate”) and pause duration in a small cohort of participants. Her analysis revealed a significantly high negative correlation between speech rate and pause duration, indicating that a slower speech rate was associated with longer pauses. In contrast, no significant relation was found between articulation rate and silent pause duration or frequency. This finding suggests that speech rate does not solely pertain to a pure rate of speaking, but includes distinct sub-processes occurring while speaking, involving articulation and the time employed in speech planning, as reflected by pause duration. More importantly, Goldman-Eisler [40] indicated that articulation rate appears to be a stable measurement in terms of variability compared to pause duration, both across participants in distinct narrative tasks and among each participant’s utterances in narration flow. Specifically in her data, pause duration exhibited a variability that was “five times higher” than articulation rate, indicating that variability in speech rate is primarily driven by silent pause of longer duration that have been related with more complex cognitive functions and not by the motor organization of speech, as reflected by articulation. The significant correlation between speech rate and pause mean time confirms the aforementioned interpretation. In cases of reduced pause duration, the articulation rate will be closer to overall speech rate, while in cases of increased pause duration, articulation rate and speech rate values will be estranged. Thus, pauses seem to have little or no direct impact upon the rate of speech movements. These findings support the initial hypothesis that pauses of longer duration serve cognitive functions in communication flow rather than articulation movements, as previously mentioned.

To our knowledge, only a few studies have explored the distinction between speech and articulation rate, as well as the impact of silent pauses on speech rate, and only in clinical populations. Cordella and colleagues [12] suggested that among speech rate, articulation, and silent pauses, only articulation rate can serve as a biomarker capable of successfully distinguishing between the three variants of primary progressive aphasia (PPA). In a more recent study from our team, we indicated that silent pauses and speech measures can successfully distinguish the three variants of PPA among themselves and from healthy speakers in two distinct narrative tasks [14]. Interestingly enough, measures derived from the picture description task appeared more sensitive compared to those extracted from the narration of a personal story. On the other hand, DeDe and Salis [8] adopted a similar approach to examine fluency measures, including speech rate, articulation rate (referred to as pure word rate in their study), and silent pause duration in a fairy tale narration from two distinct groups of patients with post-stroke aphasia (one with latent aphasia and another with anomic deficits). Their findings showed reduced speech rate and increased silent pause duration in both aphasia groups compared to controls. However, no significant differences were found between the anomic and latent aphasia sub-groups. As previously shown in Cordella and colleagues [12], for patients with PPA, articulation rate was decreased in both patient groups compared to healthy speakers but was also significantly decreased in the anomic group when compared to the latent aphasia group. While no further exploration of the relationships between the three fluency measures was attempted, the authors acknowledge the need for additional studies investigating pause patterns in patients with mild language deficits resulting from stroke.

### 1.3. The Importance of Assessing Different Speech Genres

The importance of exploring connected speech across various speech genres has been well documented in the existing literature. Studies have shown that performance across several microlinguistic and macrolinguistic measures of speech output can vary depending on the elicitation technique used [2,41,42], suggesting that different speech genres entail distinct cognitive tasks, incorporating varied levels of difficulty [43]. Intriguingly, it has been proposed that each speech genre represents a unique cognitive task associated with separate networks of cortical and subcortical brain areas [7,9].

Ulatowska and colleagues [42] have highlighted that both individuals with aphasia and healthy speakers exhibit distinct behaviors concerning morphosyntactic aspects of speech, such as the use of embedding, and the content and clarity of expression in tasks involving procedural and narrative discourse. In addition, there is evidence related to verbal patterns, including lexical diversity and verbal production, observed in both patients with aphasia [44,45,46] and healthy speakers [45].

Richardson and Dalton [47] have investigated the occurrence of main concepts in a picture sequence narrative task, a traditional story narration, and a procedural narrative task using a large sample of healthy speakers. Their findings revealed distinct age-related patterns in different narrative tasks, specifically story narration and procedural speech. Similarly, Capilouto and colleagues [5] observed that a younger group of healthy individuals generated a significantly higher number of words in a series of pictures compared to single picture descriptions. However, no such differences were identified for metrics of informativeness, lexical diversity, syntactic complexity, and main events.

With regard to neural correlates of connected speech, there is evidence from clinical populations that language measures from distinct tasks may be related to different brain regions. Efthymiopoulou and colleagues [9] observed that speech rate in a free narration task, derived from speech samples of a cohort of patients with post-stroke aphasia, was significantly correlated with lesions in the frontotemporal extreme capsule fasciculus. This white matter tract in the ventral stream is associated with selectively retrieving information from long-term memory [48,49]. Interestingly enough, no such correlation was detected for speech rate in a picture description task. The authors suggest that this dissociation pattern provides evidence supporting the hypothesis that each elicitation technique reflects a distinct cognitive process with specific demands, engaging specific brain networks.

### 1.4. Aim of Current Study

Summing up the discussion above, the existing evidence on connected speech is limited and primarily stems from studies focused on specific linguistic measures. Further research spanning a range of linguistic measures is imperative in order to gain a comprehensive understanding of the distinguishable aspects of speech production across various speech genres. Moreover, comparative studies directly investigating healthy speakers’ ability across distinct narrative tasks is limited, while no evidence exists for Greek.

The aim of the present study is to propose a methodological pipeline for the processing, quantification, and analysis of connected speech with the use of specialized linguistic software. This study specifically focuses on the examination of silent pauses, speech rate, and articulation rate as crucial components of interest. To expound on the application of the proposed methodology, we showcase the results obtained from the analysis of speech samples gathered from healthy speakers performing two distinct tasks, namely a picture description and a personal story narration.

## 2. Suggested Methodological Pipeline for the Connected Speech Analysis Protocol (CSAP)

### 2.1. Orthographical Transcription

The recorded speech samples have to undergo orthographic transcription, adhering to the fundamental conventions of discourse analysis transcription (for Greek, see [50]). Additionally, the coding conventions from the Greek Aphasia Error Corpus, as detailed by Kasselimis et al. [51], have to be applied during transcription. Instruction by examiners and extraneous discussion between examiners and participants that are irrelevant to the narration content should be identified and subsequently omitted.

### 2.2. Audio Files Prepossessing

**Figure 1:**
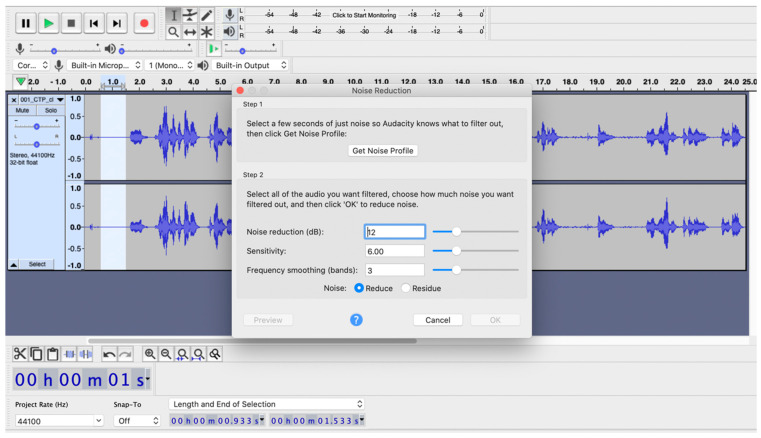
Healthy speaker’ speech sample of “Cookie theft” picture description. Noise reduction using Audacity, following specific parameters based on the specific audio file’s noise characteristics.

The audio files are going to undergo initial preprocessing using Audacity (https://www.audacityteam.org), an open source, cross-platform audio software (see Figure 1). This step aims at diminishing audio noise and eliminating non-narrative segments (as previously mentioned for the transcription part). Subsequently, the precise duration of each participant’s narrative task will be extracted in milliseconds. New audio files will then be generated. To ensure uniformity in the number of words for subsequent analysis, speech samples can be standardized to a specific word count, for instance the initial 100 words uttered. This reduction in the number of words aligns with previous linguistic and temporal analyses [3,22], while it is also in accordance with the “Quantitative Production Analysis” protocol ([52,53] for an updated version), adapted in Greek [54].

### 2.3. Silent Pauses and Speech Annotation

**Figure 2:**
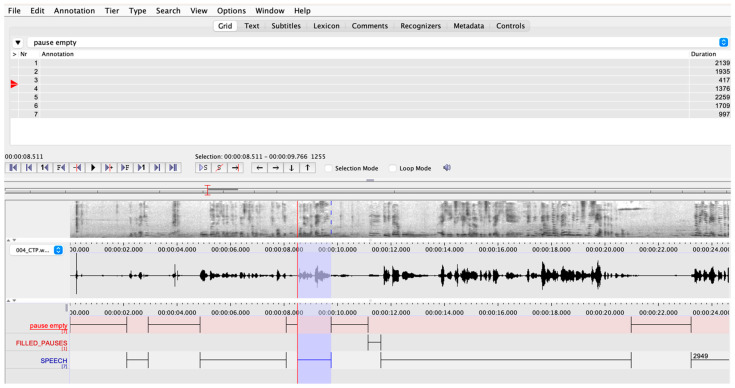
Healthy speaker’s speech sample of cookie theft picture description. Sample of multi-tier speech annotation made in ELAN. Tiers included: Empty Pauses (silent pauses), Filled Pauses (vocalized pauses such as “um”, “uh”, “hmm”), Speech (narration uttered). Purple indicates a specific section of audio file from speech output wave.

Temporal aspects of speech, encompassing the duration of both silent and filled pauses, along with speech phonation time will be annotated using ELAN program [55,56]. ELAN, a professional annotation tool designed for processing audio and video data, facilitates annotations in distinct tiers (see Figure 2).

**Figure 3:**
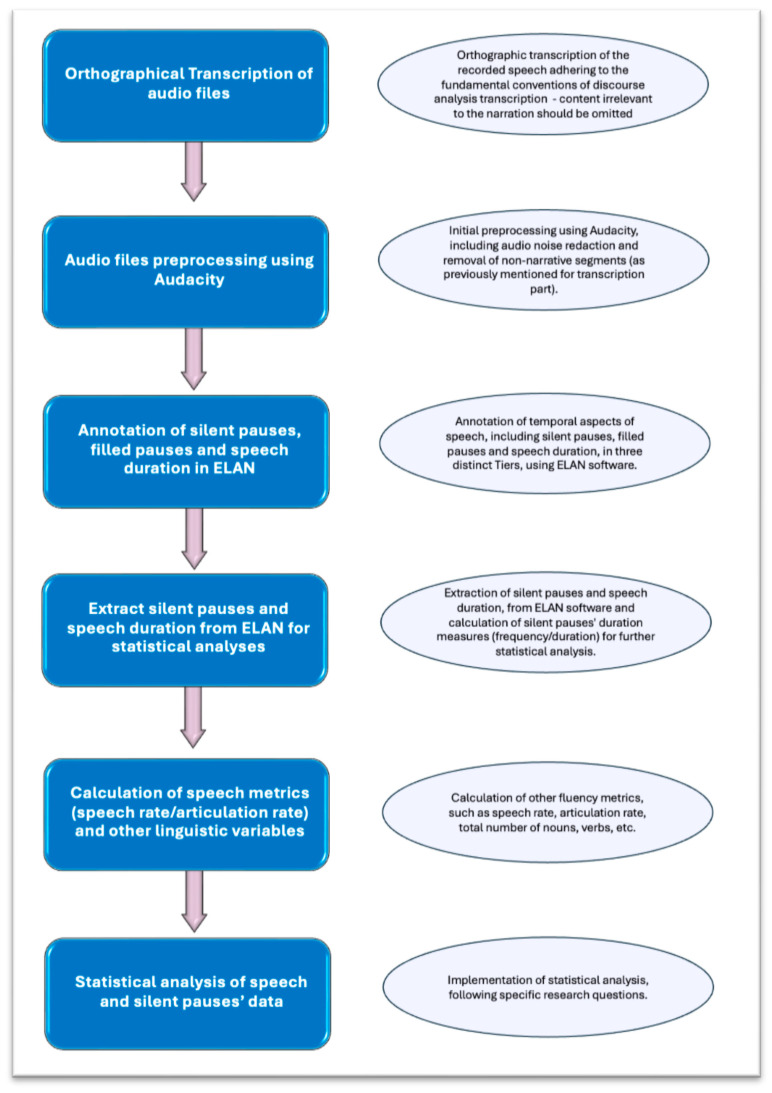
Flow chart indicating connected speech pipeline processing, from initial steps of recorded audio files such as subscription, until final steps of calculation of speech metrics. Initial steps refer to all audio files regardless of specific research questions, while final steps depend on the exact research questions of each study.

In our study, three tiers will be established to independently annotate empty pauses, filled pauses and speech, delineating the time allocated to phonation (see Figure 3 for a multi-tier annotation sample). It is noteworthy that filled pauses will be annotated solely for subsequent removal from further analysis, given their perceived distinct functional role in oral speech production [57]. Moreover, considering that numerous participants do not produce any filled pauses, their exclusion becomes particularly relevant. This approach is crucial since a combination of silent and filled pauses has often been observed in several studies complicating the interpretation of pauses’ significance in speech.

All detectable pauses will be included in the annotation process, employing two criteria: the absence of any produced speech sound produced and visual inspection of waveform, following the methodology outlined by Angelopoulou and colleagues [22]. In essence, no a priori thresholds are applied in the annotation of silent pauses [22].

Following the completion of annotation, various variables can be computed for each participant, including individual values of silent pause duration, total duration, mean and median duration of silent pauses, and individual standard deviation, all measured in milliseconds. Subsequently, all these values undergo a transformation into logarithmic values using the natural logarithm ln, as outlined in the work by Angelopoulou et al. [22] following Campione and Véronis [32]. They have indicated that the log-normal distribution offers a significantly improved alignment with the data compared to raw data distribution, thus statistical analyses should be conducted in the logarithmic domain, casting uncertainty on the findings of numerous studies mentioned in the literature that employ the arithmetic domain. Additionally, the total frequency of silent pauses, the frequency of silent pauses occurring prior to nouns and verbs, and the overall count of open class words can also be calculated, as they consist of words with semantic content, thus investigation of silent pauses before them may perceived as indices of cognitive processing such word finding and retrieval. Furthermore, the frequency of silent pauses between and within utterances can also be determined, aligning with specific research questions (refer to Table 2 for a comprehensive presentation of temporal and linguistic variables).

### 2.4. Further Linguistic Variables Annotation

Further linguistic analysis can also be conducted in speech samples including the calculation of the total number of open class words (that is content words having semantic content, including nouns, verbs, adjectives, and adverbs), the total number of nouns, the total number of verbs, and the annotation of clause-like units. A clause-like unit (CLU) is defined as a syntactically and/or prosodically marked stretch of speech containing one verb, and more specifically a clause is assumed syntactically complete when a verb and all its arguments are apparent (see [58,59]). It should be noted that due to the increased confounds in literature with respect to utterance annotation (see [60], for an extended discussion), it has been decided to annotate speech on the basis of the simplest definition of clause, as presented above, following Goldman-Eisler [29] and Grande et al. [59], and not by using a definition for the utterance. The main aim is to annotate whether silent pauses occurred between or within clauses.

Speech rate is calculated by dividing the total number of syllables with the total duration of speech (including all disfluency measures, namely silent and filled pauses) [(number of syllables × 60 s)/total duration]. Articulation rate is calculated by dividing the total number of syllables with the duration of phonation time (excluding all disfluency measures) [(number of syllables × 60 s)/duration of phonation]. It should be noted that for both metrics we used the number of syllables rather than number of words to extract more accurate measurements. This approach is also followed by Themistocleous et al. [18]. See Figure 3 for a summary of the presented pipeline.

## 3. An Example of the Implementation of CSAP

### 3.1. Participants

To test our suggested methodology, we used speech sample data from sixty-five healthy individuals (thirty-three males), 25–65 years old (mean: 44.46, SD: 11.82) and 9–18 years of formal schooling (mean: 15.37, SD: 3.19), recruited in the frame of the project “Investigation of cortical surface patterns and their relation with speech metrics and performance in neuropsychological assessment in healthy participants” conducted in the Neuropsychology and Language Disorders Unit of the First Neurology Department, School of Medicine, National and Kapodistrian University of Athens, Eginition Hospital (research protocol approval ID: ΩOΞΛ46Ψ8N2-7PN, July 2017). Informed consent was obtained from all participants prior to participation, according to the Ethics Committee of Eginition Hospital. All participants were right-handed, monolingual Greek speakers, and permanent residents of Athens. Participants with a history of neurological and/or psychiatric disorders were excluded from the study (see also [3]).

### 3.2. Speech Samples

A personal story narration and “cookie theft” picture description were acquired from each participant during individual examination, as part of the Boston Diagnostic Aphasia Examination standard assessment (BDAE-SF) [61], adapted for Greek [62]. More specifically, for the personal story, participants were asked to describe a medical event of their own or of someone related to them, as a recount of a past event, a narration equivalent to the stroke story (see [3,22]). For the cookie theft picture (included in the BDAE-SF) description, patients were asked to describe everything they can see in the picture. No time restrictions were applied, and participants were free to speak for as long as they wanted in both tasks. In the case of exceedingly brief narrations and/or the occurrence of long silences, the examiner provided minimum encouragement, through a standard set of questions, based on specific instructions.

### 3.3. Statistical Analysis

In order to test our methodology, we calculated speech rate, articulation rate, silent pauses frequency, and silent pauses total duration adjusted for total duration of narration, in the two narrative tasks. First, we compared the aforementioned speech and silence measures across the two narrative tasks. For that reason, we conducted separate mixed effects models (LMM), entering the narrative task as fixed factor and participants as random factor. Then, we investigated whether silent pauses’ metrics, i.e., frequency and duration, presented any significant correlations with articulation rate and speech rate. For that purpose, Pearson r correlation analyses were conducted.

All statistical analysis was conducted using the open-source statistical package R (R Development Core Team, 2011) (http://www.r-project.org), using the using the lme4 package (R package lme4) [63], implementing the Satterthwaite approximations for degrees of freedom [64]; see also [65] for more details). All plots were conducted using the ggplot2 package [66] for R (R Core Team 2015).

### 3.4. Results

**Figure 4:**
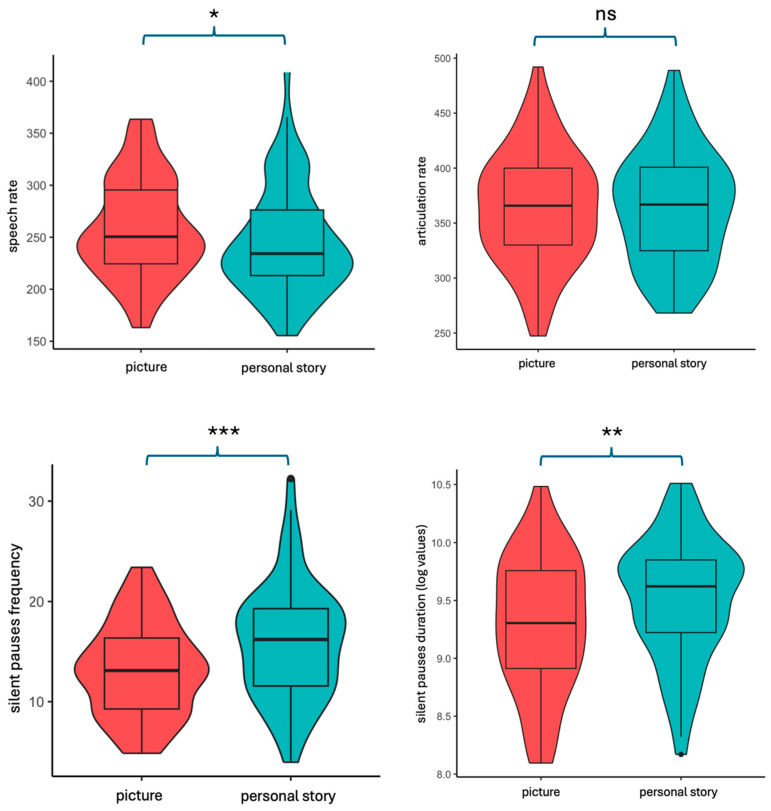
Violin plots including error bars and box plots, showing the distribution of the data, during the two discourse genres. Straight black bold line indicates the mean.

Results indicated that speech rate is significantly increased in the picture description task [t (64) = −2.02, *p* = 0.0476], while pause frequency and pause total duration are significantly increased in the patient story narrative condition [t (64) = 3.814, *p* = 0.00031 and t (64) = 3.351, *p* = 0.00135, for pause frequency and duration, respectively]. Articulation rate showed no significant differences between the two narrative tasks (see Figure 4).

To explore the influence of silent pauses on articulation rate and speech rate, separate correlation analyses were performed for each narrative task, examining the frequency of silent pauses, total duration of silent pauses, speech rate, and articulation rate. Normality tests using the Shapiro–Wilk test showed no statistically significant deviations for any linguistic variable, allowing the use of the Pearson correlation coefficient.

For the picture description condition, the analyses indicated that articulation rate did not exhibit a significant correlation with any measures of silent pauses (frequency or duration). In contrast, correlation analyses involving speech rate demonstrated a significant negative correlation between silent pause total frequency [r(65) = −0.617, *p* < 0.001] and silent pause total duration [r(63) = −0.724, *p* < 0.001], indicating that more frequent and longer pauses are associated with a lower speech rate.

**Figure 5:**
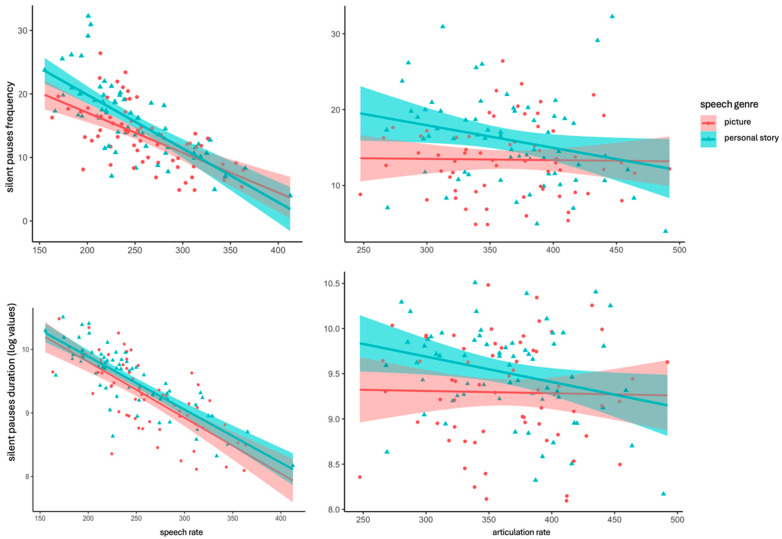
Scatter plots indicating negative correlations between silent pauses metrics (frequency and total duration) and speech rate, and no correlations with articulation rate in picture description (pink) and personal story (green).

Similarly, in the personal story narration, correlation analyses revealed that articulation rate did not present a significant correlation with any of the silent pauses’ measures (frequency or duration). However, correlation analyses including speech rate presented a negative high correlation between silent pause total frequency [r(65) = −0.731, *p* < 0.001] and silent pause total duration [r(63) = −0.827, *p* < 0.001], suggesting that more frequent and longer pauses are related with lower speech rate, as previously mentioned for picture description (see Figure 5).

## 4. Discussion

In the current study we introduced a methodological approach for analyzing connected speech. Our aim is rooted in the realization that previous studies have followed an array of different methodological decisions concerning the calculation of speech and silent pause measures, and so has their subsequent analysis. This variability in approaches raises several issues, making the results of different studies incomparable and meta-analysis challenging.

Concerning silent pauses, we initially annotated all detectable silent pauses in speech flow without employing any predetermined threshold. This involved annotating all silent pauses, following the suggestion of Campione and Véronis [32], using ELAN, as described in the Methods Section. By doing so, we avoided the exclusion of specific pauses with a duration of less than 200 ms, a practice seen in previous research (e.g., [15,16,17,24,27]). It should be noted that in our data analysis example, we did not proceed with the investigation and calculation of a threshold. However, in such cases, a posteriori analysis in each dataset would reveal whether a threshold actually exists, as indicated in a previous study [22]. Previous findings on a posteriori threshold calculation suggest that a potential threshold separating pauses based on their duration is considered “dynamic”, influenced by several factors, including speaker health, age, sex, and the genre of the elicitation technique [33]. It is important for researchers to bear this in mind for future studies that aim at developing complete and accurate datasets and, consequently, reliable results.

In our methodological approach, we recommend conducting an investigation into both silent pause frequency and duration. As discussed in the introduction, previous studies have often reported either frequency or duration separately, while there is no consensus in the formulation of these measures. We emphasize the importance of examining both frequency and duration, as they represent two distinct metrics that may reflect different cognitive processes during speech. As we have highlighted in a previous study [14], the frequency of pauses denotes how often a speaker stops and may be related with various cognitive processes such as processing new information or revising the previously spoken utterance or may reflect the cognitive exertion required in order to execute the intended utterance originally planned. Moreover, it has been suggested that the number of times a speaker stops may function as a compensatory mechanism, especially in cases of word finding deficits (see, for example, [16,17,24]). Silent pause duration, on the other hand, may reflect the level of efficacy and the amount of time that each individual needs in order to accomplish such cognitive processes while speaking [14]. Therefore, we emphasize the need for further clarification regarding the potentially distinct roles of silent pause frequency and duration and their connection to internal cognitive processes. This involves the use of specific metrics to achieve homogeneity in results, facilitating comparisons of research findings and meta-analyses. Furthermore, we propose the use of specific measures, by creating an index for the number of silent pauses normalized by the total number of words. Conversely, when addressing the duration of silent pauses, we recommend using the total duration of pauses normalized by the total duration of speech output. We posit that total duration may offer more informative insights compared to mean or median values.

In our current methodological approach, we have excluded filled pauses from further analysis, aligning with the approach taken in previous studies (e.g., [13,14,15,22,23,25]). We emphasize the importance of continuing to treat them as distinct entities, at least until we can elucidate the exact roles of both categories of pauses. We believe that considering them as the same linguistic phenomenon (as seen in, for example, [24,27]) may lead to confounding results. For instance, Salis and Dede [27] combined the durations of both silent and filled pauses, asserting that both types indicate hesitation phenomena associated with planning and monitoring processes, as proposed by Levelt [67]. Whereas Levelt [68] has suggested that pauses may serve as editing phenomena, recent studies have not precisely clarified the distinct roles of silent and filled pauses. Zellner [57] introduced them as two distinct phenomena based on their definitions. However, empirical data, including our dataset, indicate that not all speakers, whether patients or individuals without neurological or psychiatric conditions, exhibit filled pauses, and when they do, these are assumed to be significantly reduced compared to silent pauses. Thus, until specific hypotheses are tested regarding their roles and functions, we recommend treating them differently.

Our results have indicated that pause metrics (frequency and duration) and speech rate significantly differ in distinct conditions of narration, personal story, and picture description, while articulation remains constant. Healthy speakers presented an increased speech rate for the picture description condition, while producing significantly increased pauses of longer duration in the personal story condition. Our findings raise two important issues regarding healthy individuals’ ability to produce oral language, namely the issue of stability versus variability of various linguistic measures of fluency in separate elicitation tasks and the distinction in definitions of such speech fluency metrics as speech rate and articulation rate and the potential effect of silent intervals in speech flow on them. Our findings support the hypothesis of the speech rate and articulation rate interrelation and how these can be affected by silent pause duration in formulation of speech output. As was discussed in the introduction, Goldman-Eisler [40,69] provided initial evidence, based on a very small cohort of speakers, regarding the independence of articulation rate from silent intervals in speech and its stability in different conditions of narration. Speech rate, on the other hand, is formulated by two independent factors, articulation and hesitations, and thus seems to be influenced by the amount of time spent on hesitations, presenting increased variability according to speech genre. It should be emphasized, however, that these findings were based on a very small sample size of participants, with no further information regarding demographics or health condition, and thus cannot be easily generalized.

Surprisingly enough, these quantitative markers of speech production have not been extensively investigated in healthy speakers, as most studies have almost solely focused on the demographics effects of speech fluency metrics, using either speech rate or articulation rate (see, e.g., [3,4,36]), while sparse evidence comes from the comparison of distinct speech genres. For instance, Ardila and Rosseli [4] investigated oral speech patterns in a large cohort of healthy speakers, using the “Cookie Theft” picture description task, while they only considered the total number of words as a production index, without accounting for time necessary for production. Regarding pause patterns, no studies so far exist directly comparing pause frequency and duration in different narrative tasks in healthy speakers. Thus, the only evidence derives from studies in patients with acquired language disorders, in which healthy speakers of small cohorts are sometimes used as a control group (see, e.g., [17,22,24]); however, even in these cases, only one elicitation task is used. Moreover, pause indices are studied in the framework of specific research questions, in which not enough evidence is provided about the major characteristics of pauses, including frequency and duration. For instance, Mack and colleagues [24] investigated silent and filled pauses in speech samples derived from patients with primary progressive aphasia but focused only on pauses appearing before nouns and verbs. As a result, the only information available refers to differences and similarities of these fluency measurements between several patient cohorts and neurotypical speakers.

Our finding concerning the difference of pauses in distinct narrative conditions may offer additional evidence in favor of the hypothesis that silent intervals may reflect cognitive processing while speaking. Considering different narrative genres as distinct tasks of different cognitive and linguistic demands [43], we would expect pauses to appear differently with respect to their frequency and duration. These characteristics have long been considered to define the role and function of pause in speech flow [29]. It has been hypothesized that more pauses of longer duration would appear in sentence/clause boundaries, reflecting the effort of sentence planning and organization. In our data, more pauses do indeed appear in the personal narration story, which is assumed to be a narrative task of increased cognitive demand, as compared to the relatively simple picture description task, at least for healthy speakers. Combining the differences between pauses with articulation ability, we could suggest that healthy individuals articulate in a similar way, regardless of speech content. Nevertheless, they consume different time to process and organize the content or perhaps even the structure of what they are planning to say, according to the elicitation task.

This raises the important issue of quantifying speech output in language studies of not only neurotypical speakers but also of patients with acquired language disorders. Our results seem to verify that speech rate is a complex index that does not clearly indicate articulation ability, as it also incorporates the amount of time that speakers use to perhaps plan and organize the content of speech, as reflected by pause occurrence. However, even in the aphasiology literature, evidence on distinguished measures of speech and articulation rate is sparse (for a discussion, see [12] for patients with primary progressive aphasia and [8] for patients with post-stroke aphasia), as most studies provide either one or the other metric [6,9,10,11], not taking into account the possibility that they reflect different aspects of speech. Thus, providing a clear definition of such speech metrics, along with a disintegration of speech rate as a metric of speech, consisting of silent pauses occurrence and articulation, is of great importance to the language study of both healthy speakers and patients with acquired language disorders.

In sum, taking into account Goldman-Eisler’s [40] early findings that speech rate is primarily affected by pause variability while articulation rate remains stable, at least in healthy speakers, in combination with later findings that narration types fulfil distinct cognitive tasks, we can assume that the differentiation of pauses in speech genres serves as an additional argument in favor of their role in speech planning and organization. In other words, pauses may reflect different internal cognitive functions occurring during speech in various situations, affecting speech rate index, while articulation rate remains constant regardless of speaking condition. However, existing evidence is limited, allowing us to suggest that it would be useful to confirm earlier hypothesis on whether the duration of silent pauses is different across speech genres, in either healthy speakers or patients with acquired language disorders. More studies are necessary in order to expand and elaborate on the hypothesis, previously presented, that the occurrence of pauses is related to linguistic and cognitive features in oral language.

Our findings provide additional evidence in favor of the general, but not extensively studied, notion that different speech genres consist of distinct cognitive tasks of varying linguistic demands [2,41,42,43]. Therefore, performance in several micro- or macrolinguistic measures of speech output may be differentiated according to the elicitation technique implemented [41]. In our study, we used two of the most common, yet completely different, elicitation techniques, widely implemented in language research of healthy individuals and patients with acquired language disorders: a single picture description and a personal story narration of an illness/accident event, equivalent to a stroke story interview for patients with aphasia (see also [22]). Picture description tasks are considered to be of low cognitive challenge, as illustrations tend to enhance discourse structure, at least for healthy individuals. As speech output and semantic content in picture description is based, almost exclusively, on visual stimuli, they are related more to aspects of cognition, such as access to lexical/semantic representations and semantic knowledge along with organization of visual content, and less on selective retrieval of information from semantic and episodic memory (see [70]). Spontaneous speech based on free narration, on the other hand, is assumed to be a distinct task related with several interactive aspects of cognition, and thus has been extensively investigated, not only in linguistics but also in cognitive psychology (for a review, see [71]). A narrative task can be considered a fulfilled description of actions and events that have evolved over time, and thus its production has been generally characterized as a challenging process that can only be successfully accomplished in cases that individuals do not confront cognitive deficits [71,72]. However, despite the significant importance of investigating language production with various elicitation techniques [2], evidence comparing the performance of healthy speakers in distinct narrative tasks is sparse.

In conclusion, evidence is so far sparse and comes from studies of individual linguistic aspects. Further research in a variety of linguistic measures is clearly necessary in order to better understand the aspects of speech production that can be differentiated in speech genres. Similarly, comparative studies directly investigating the performance in distinct narrative tasks in healthy speakers, using certain qualitative or quantitative aspects of speech, are also needed. It can be argued that oral language production, under different circumstances, is an important issue that pertains to several scientific fields, such as neuropsychology and neurolinguistics, while it has a major purport for the investigation of language disorders, as regards both assessment and treatment. The analysis of language samples has been gaining in prominence among clinicians and researchers, since it offers a unique chance to directly observe a variety of complex cognitive and linguistic behaviors. Nevertheless, the implementation of different narrative tasks, including picture description, personal story narrations, as well as procedural discourse or recall and narration of well-known stories, can be used to further investigate several silence and fluency variables.

## 5. Conclusions

Our suggested methodological approach offers a comprehensive framework for investigating speech output patterns, incorporating fluency metrics and silence measures, while the use of distinct narrative tasks can enhance our understanding of connected speech in both healthy and clinical populations. We emphasize the significance of adopting a unified methodological approach in connected speech studies, enabling the integration of results for more robust and generalizable conclusions. The proposed CSAP, which we have already implemented in speech samples of healthy participants [3] as well as in patients’ cohorts with post-stroke aphasia [7,22] and primary progressive aphasia [14] is offered as a relevant tool to researchers in the field, providing a standardized and effective means to examine connected speech across diverse populations and distinct speech genres. This approach not only addresses existing methodological inconsistencies but also facilitates the synthesis of findings for a more cohesive understanding of speech production processes.

## Figures and Tables

**Table 1:** Microstructure linguistic elements derived from connected speech analysis in contemporary research.

Microstructure Indices		
Speech Metric	Description	References
**Speech rate**	Words (or syllables) per minute	**Healthy speakers** Angelopoulou et al., 2020 [3] Ardila and Rosselli, 1996 [4] (*referred as total number of words*) Capilouto et al., 2016 [5] (*referred as total number of words*)
		**Patients with post-stroke aphasia** Andreetta et al., 2012 [6] Angelopoulou et al., 2024 [7] Dede and Salis, 2020 [8] Efthymiopoulou et al., 2017 [9] Fromm et al., 2017 [10] Gordon and Clough, 2020 [11]
		**Patients with primary progressive aphasia** Cordella et al., 2017 [12] Nevler, et al., 2019 [13] Potagas et al., 2022 [14]
		**Patients with MCI/AD** Lofgren and Hinzen, 2022 [15] Pistono et al., 2016; 2019 [16,17] Themistocleous et al., 2020 [18]
		**Patients with psychiatric disorders** Cohen et al., 2014 [19] (*referred as total number of words*)
**Articulation rate**	Words (or syllables) per minute, referring only to phonation time	**Healthy speakers** Robb et al., 2004 [20]
		**Patients with post-stroke aphasia** Dede and Salis, 2020 [8]
		**Patients with primary progressive aphasia** Cordella et al., 2017 [12] Potagas et al., 2022 [14]
		**Patients with MCI/AD** Themistocleous et al., 2020 [18]
		**Patients with psychiatric disorders** Hampsey et al., 2022 (*a suggested study*) [21]
**Silent pauses’ frequency**		**Patients with post-stroke aphasia** Angelopoulou et al., 2018; 2024 [7,22]
		**Patients with primary progressive aphasia** Baqué et al., 2023 [23] Cordella et al., 2017 [12] Mack et al., 2015 [24] (*restricted to nouns and verbs*) Nevler, et al., 2019 [13] Potagas et al., 2022 [14]
		**Patients with MCI/AD** Lofgren and Hinzen, 2022 [15] Pistono et al., 2016; 2019 [16,17]
		**Patients with psychiatric disorders** Cohen et al., 2014 [19] Çokal et al., 2019 [25] Rapcan et al., 2010 [26]
**Silent pauses’ duration**		**Patients with post-stroke aphasia** Angelopoulou et al., 2018; 2024 [7,22] Dede and Salis, 2020 [8] Salis and Dede, 2022 [27]
		**Patients with primary progressive aphasia** Baqué et al., 2023 [23] Cordella et al., 2017 [12] Nevler, et al., 2019 [13] Potagas et al., 2022 [14]
		**Patients with MCI/AD** Lofgren and Hinzen, 2022 [15] Pastoriza-Dominguez et al., 2022 [28] Pistono et al., 2016; 2019 [16,17]
		**Patients with psychiatric disorders** Cohen et al., 2014 [19] Rapcan et al., 2010 [26]

**Table 2:** Linguistic elements derived from analysis of the two narrative tasks.

	Speech Variables	
1.	Total duration of speech	counted in milliseconds (msec)
2.	Total duration of phonation	duration of speech minus duration of disfluencies (silent and filled pauses), counted in milliseconds (msec)
3.	Total duration of silent pauses	duration of speech minus duration of phonation and filled pauses), counted in milliseconds (msec)
4.	Mean duration of silent pauses	calculated from the total cohort of individual pauses for each participant
5.	Median duration of silent pauses	calculated from the total cohort of individual pauses for each participant
6.	Total number of syllables	
7.	Total number of words	
8.	Speech rate	number of syllables to total duration of speech [(total number of syllables × 60)/total duration of audio file]
9.	Articulation rate	number of syllables to total duration of phonation [(total number of syllables × 60)/duration of phonation]
10.	Pause frequency	total number of pauses to total number of words [(total number of silent pauses × 100)/total number of words]
11.	Pause frequency between sentences	total number of pauses between sentences to total number of words [(total number of silent pauses between sentences × 100)/total number of words]
12.	Pause frequency within sentences	total number of pauses within sentences to total number of words [(total number of silent pauses within sentences × 100)/total number of words]
13.	Open class word frequency	total number of open class words to total number of words [(total number of open class words × 100)/total number of words]
14.	Pause frequency before open class words	total number of pauses before open class words to total number of open class words [(total number of silent pauses before open class words × 100)/total number of open class words]
15.	Noun frequency	total number of nouns to total number of words [(total number of nouns × 100)/total number of words]
16.	Pause frequency before nouns	total number of pauses before nouns to total number of nouns [(total number of silent pauses before nouns × 100)/total number of nouns]
17.	Total number of verbs	total number of verbs to total number of words [(total number of verbs × 100)/total number of words]
18.	Pause frequency before verbs	total number of pauses before verbs to total number of verbs [(total number of silent pauses before verbs × 100)/total number of verbs]
19.	Number of clause-like units (CLU)	a syntactically and/or prosodically marked part of speech containing one verb

## Data Availability

Data are unavailable due to privacy and ethical restrictions.
